# Prescribing and medical non-adherence after myocardial infarction: qualitative interviews with general practitioners in Germany

**DOI:** 10.1186/s12875-020-01145-6

**Published:** 2020-05-08

**Authors:** Christian Freier, Christoph Heintze, Wolfram J. Herrmann

**Affiliations:** 1 Charité – Universitätsmedizin Berlin, corporate member of Freie Universität Berlin, Humboldt-Universität zu Berlin, and Berlin Institute of Health, Institute of General Practice, Charitéplatz 1, 10117 Berlin, Germany; 2 Münster School of Health, FH Münster - University of Applied Sciences, Leonardo Campus 8, 48149 Münster, Germany

**Keywords:** Family practice, General practice, Primary care, Myocardial infarction, Secondary prevention, Prescribing, Medication adherence, Qualitative research, Interviews

## Abstract

**Background:**

An increasing prevalence of having survived a myocardial infarction increases the importance of medical secondary prevention. Although preventive medication reduces mortality, prescribing and adherence are known to be frequently insufficient. General practitioners are the most important prescriber. However, their perspective on prescribing and medical non-adherence following myocardial infarction has not yet been explored. Thus, the aim of this study was to explore the general practitioners’ perspective on long-term care after myocardial infarction focussing on medical prevention.

**Methods:**

In this qualitative interview study we conducted episodic interviews with sixteen general practitioners from rural and urban surgeries in Germany. Framework analysis with focus on general practitioners’ prescribing and patients’ non-adherence was performed.

**Results:**

Almost all general practitioners reported following guidelines for myocardial infarction aftercare and prescribing the medication that was initiated in the hospital; however, they described deviating from guidelines because of drugs’ side effects or patients’ intolerances. Some questioned the benefits of medical secondary prevention for the oldest of patients.

General practitioners perceived good adherence among their patients who had had an MI while they regarded their methods for assessing medical non-adherence as limited. They perceived diverse reasons for non-adherence, particularly side effects, patients’ freedom from symptoms and patients’ indifference to health. They attributed mainly negative characteristics, like lack of knowledge and understanding, to non-adherent patients. These characteristics contribute to the difficulty of convincing these patients to take medications as prescribed.

General practitioners improved adherence by preventing side effects, explaining the medication’s necessity, facilitating intake and involving patients in decision-making. However, about half of the general practitioners reported threatening their patients with negative consequences of non-adherence.

**Conclusions:**

General practitioners should be aware that discharge medication can be insufficient and thus, should always check hospital recommendations for accordance with guideline recommendations. Improving physicians’ communication skills and informing and motivating patients in an adequate manner, for example in simple language, should be an important goal in the hospital and the general practitioner setting. General practitioners should assess patients’ motivations through motivational interviewing, which no general practitioner mentioned during the interviews, and talk with them about adherence and long-term treatment goals regularly.

## Background

Globally, coronary heart disease (CHD) accounted for 8.93 million deaths in 2017 [1]. The incidence of myocardial infarction (MI) is declining in Germany and the USA [2, 3]; however, the case fatality rate is also decreasing, with an increasing percentage of patients surviving MI in Europe and the USA [3, 4].

Secondary preventive medication significantly reduces MI survivors’ mortality [5, 6]. According to current guidelines for both ST-elevation myocardial infarction (STEMI) [7, 8] and non-ST-elevation myocardial infarction (NSTEMI) [9, 10], aspirin and statins are recommended and beta-blockers and angiotensin converting enzyme inhibitors (ACEI) should be considered, each unless there are contraindications. Angiotensin receptor blockers (ARB) are an alternative to ACEI [7–10]. P2Y_12_ inhibitors for dual antiplatelet therapy (DAPT) are usually recommended for twelve months [7–10].

General practitioners (GPs) play an important role in long-term care after MI [11–14]. In Germany 97% of patients visited a GP in the fourth quarter after their MI while only 23% visited an office-based cardiologist [14]. However, only 44% of these patients had prescriptions for all four drug groups (aspirin and P2Y_12_ inhibitors as one group, ACEI and ARB as one group) filled in the quarter of their MI, decreasing to 24% in the fourth quarter after MI [14]. Furthermore, it has been shown that outpatient prescribing of the recommended drugs following MI is suboptimal [15–19]. Questionnaire studies with GPs explored reasons for not prescribing these drugs, including side effects, intolerances and contraindications [20–22]. However, these studies were not open for emerging reasons which were not addressed by the questionnaires [20–22]. In one of these studies about prescribing of beta-blockers the preparation of the questionnaire included, among other sources, interviews with physicians, but it has not been shown which items they contributed to the final questionnaire [20]. Additionally, some qualitative studies with GPs focused on the prescribing of statins [23–25], but none of them focused on secondary prevention and there is a lack of qualitative studies addressing the prescribing of the other recommended drugs.

Known factors associated with low prescribing rates are the type of MI [19, 26] as well as the gender [15, 27] and age of patients [15, 16, 22, 27, 28]. Prescribing seems to be lower in patients after NSTEMI [19, 26], but reasons for this are unknown. Prescribing is also lower in women after MI or coronary revascularisation than in men [15, 27]. Women reported more frequent side effects under statins [29], and their antiplatelet therapy has been more frequently stopped in case of bleeding after MI [30]. A strong factor in gender disparities around statin prescription in CHD was women’s higher age [14, 29]. The prescribing of secondary preventive medication is also lower in older patients [15, 16, 22, 27, 28]. Qualitative research investigating reasons for this gap in secondary prevention is limited to a study with GPs from the Netherlands, which revealed uncertainties relating to guidelines, doctors, patients and organisation [31]. Comorbidities may lead to deviation from guidelines since guidelines rarely address such complexities [32].

Non-adherence to medication following MI is also a known issue [33]. Knowledge of possible reasons for non-adherence enables physicians to recognise individual barriers to adherence [34, 35]. However, few studies have reported the ways in which physicians assess adherence to secondary preventive medication [36]. In addition, to our knowledge, physicians’ perceptions of reasons for non-adherence after acute coronary syndrome have only been studied for Clopidogrel in the USA and the perceived main reason were the costs for the patients [37]. However, this seems to be specific for the USA, e.g. in Germany most of the costs are covered by statutory health insurances.

As shown, GP care is crucial for medical prevention after MI; however, the GPs perspective on long-term care after MI has been unknown yet.

Thus, the aim of this study was to research the GPs’ perspective on long-term health care following MI. For this article we focus on the GPs’ perspective on prescribing and medical non-adherence as well as their attempts to improve adherence.

## Methods

This qualitative interview study is part of a larger project examining long-term care after MI in the GP setting. Between March and June 2018 we conducted face-to-face, semi-structured, episodic interviews with GPs working in the German federal states of Berlin (urban) and Brandenburg (rural).

Several recruitment strategies were applied to achieve a sample with a broad diversity [38] regarding GPs’ age and the number of physicians in each surgery and with a nearly equal distribution of GPs’ genders and of the number of surgeries located in each of the two federal states: advertising in a newsletter of the Research Network of the Berlin Institute of General Practice Charité (ANCHOR), personal letters to GPs who had recruited patients for another interview study of this project about patients’ perspectives on long-term care after MI, and asking GPs who were working in teaching surgeries of the Charité – Universitätsmedizin Berlin during a workshop. Sixteen GPs were recruited and interviewed: three GPs who responded to the newsletter, six of the ten GPs who got a personal letter and seven of the twenty-two workshop participants. Table 1 gives an overview of participants’ characteristics.

**Table 1:** Characteristics of interviewed GPs. Percentages of the whole sample in brackets

	GPs working in Berlin	GPs working in Brandenburg
Number of interviewed GPs	9 (57)	7 (44)
Gender		
Female	5 (31)	4 (25)
Male	4 (25)	3 (19)
Age (range: 38–64 years)		
35–44	2 (13)	3 (19)
45–54	3 (19)	2 (13)
55–64	4 (25)	2 (13)
Number of physicians in surgery		
1	3 (19)	1 (6)
2	2 (13)	5 (31)
3	2 (13)	0 (0)
4 or more	2 (13)	1 (6)

Based on the assumption that ‘subjects’ experiences of a certain domain are stored and remembered in form of narrative-episodic and semantic knowledge’, episodic interviews are a method to access both of these forms, enabling in-method triangulation between these [38]. ‘Episodic knowledge is [...] linked to concrete situations and circumstances’ and made accessible by prompts for case narratives [38]. Accordingly, GPs were repeatedly encouraged to narrate cases of their patients who had had an MI some time ago. Furthermore, these case narratives can balance social desirability effects. Semantic knowledge, which contains ‘concepts and their relation to each other’, ‘is made accessible by concrete pointed questions’ [38].

An interview guide (Additional file 1) covering different aspects of GPs’ long-term care for these patients was developed. It was then revised in collaboration with other qualitative researchers from our institute during a workshop after they had been informed about the aim of the study. The guide was tested in two pilot interviews and served for the interviewers’ orientation during the interviews.

The first author conducted the interviews. He was knowledgeable about long-term care after MI and received training in conducting qualitative interviews. He was in no relationship to the participants. To facilitate GPs’ participation and to make them comfortable in the interviews the GPs determined the interview settings: fifteen were conducted in GPs’ surgeries and one at a GP’s home. The interviews had a mean duration of 71 min (SD = 18 min) and were audio recorded. Context records and, if necessary, post-interview-memos were made [38].

The first author transcribed the recordings verbatim after each interview and anonymised them. A summary was written for each interview. After each transcription, the interview was searched for emergent themes and aspects of themes which matched the researched question and were not addressed by the interview guide [39]. Entirely new themes were added to the guide as new questions after emerging in three of the conducted interviews. New aspects belonging to themes which were already contained in the guide were added to the guide as new questions after emerging in one of the conducted interviews. The new questions were then applied to all remaining interviews. In the interview guide (Additional file 1) these new questions are written in italics and commented with the number of the interview to which they were applied for the first time. In the five last interviews no new themes or aspects emerged. Therefore, saturation was assumed.

To analyse the qualitative data, we applied Ritchie and Spencer’s framework analysis [40]. It addresses both a priori issues and freshly emergent themes, improves transparency [41] and allows a comprehensive review of the data [40]. By conducting the interviews, transcribing and re-reading summaries, the first author became familiar with the data and set up a thematic framework. The first and the last author consecutively elaborated the framework with each of the following steps: incorporating the interview guide; testing the framework on three of the later interviews, which varied according to GP and surgery characteristics; coding all transcripts with the software f4analyse 2.0.3 EDUCATION; reviewing the coding in a workshop with experienced qualitative researchers; final elaboration of the categories which concerned medication; and final coding of these categories. For each category concerning medication, summaries of the corresponding coded text segments for every GP were tabulated.

All participants gave written informed consent. The local ethical committee approved the study.

The first author translated the quotes into English. All quotes are marked with the gender (F = female, M = male) and the surgery location (B = Berlin, BB = Brandenburg) of the respective interviewee.

## Results

Analysis of the data regarding medical therapy after MI revealed two major topics: ‘Prescribing of medication after MI’ focuses on GPs’ prescribing behaviour; ‘Non-adherence of patients’ focuses on GPs’ perspectives on patients’ non-adherence. Table 2 shows the topics’ subthemes. In the following, we will elaborate on these topics. The subthemes’ subcodes are also listed in Additional file 2.

**Table 2:** Major topics and subthemes

**Major topic 1: Prescribing of medication after MI**	
- Prescribed medications	
- Hospital recommendations	
- Cardiologists’ role in GPs’ prescribing	
- GPs’ perspectives on the impact of NSTEMI, patients’ gender and patients' age on prescribing	
- Further reasons for not prescribing guideline recommended medication	
**Major topic 2: Non-adherence of patients**	
- GPs’ perception of non-adherence	
- Assessing medical non-adherence	
- Attributed reasons for non-adherence	
- Improving adherence	

### Prescribing of medication after MI

#### Prescribed medications

The interviewed GPs claimed to prescribe ACEI, beta-blockers, aspirin and statins for all or most patients who had had an MI, as recommended by guidelines:

*‘[...] that are always ACE inhibitor or a sartan, beta-blocker, a statin and ASS 100 [aspirin].’* (GP2, M, BB)

An exception from this was one GP who stated that he did not see any necessity for statins in patients after MI with normal lipid values.

However, when the GPs were asked about a recent consultation with a patient who had had an MI, it was revealed that almost half of the interviewed GPs had not prescribed one or two of the recommended drugs in the respective case.

#### Hospital recommendations

Almost all interviewed GPs reported that they continued prescribing the medication initiated in the hospital, with a few expressing their trust in the inpatient cardiologists:

*‘What I prescribe is recommended to me by the hospital, and then I don’t enquire properly. They are absolute experts in the hospital.’* (GP5, F, B)

Only one GP made the point that he would not adopt hospitals’ recommendations if they were nonsense, although that had never been the case, while another GP emphasised that she calls the hospital if there is an inconsistency in the discharge letters.

However, one GP complained that some patients are discharged on medication which has already been proven to be inappropriate for them. She therefore includes information about every patient’s prior medication on the admission form.

#### Cardiologists’ role in GPs’ prescribing

In Germany office-based cardiologists usually run their own surgeries, and GPs can refer patients to these cardiologists. All interviewed GPs stated that they prescribe medication for secondary prevention themselves. However, some GPs explained that it is hard to keep track of the recommendations for DAPT and triple therapy and another GP said that she is inexperienced in prescribing sacubitril/valsartan combination or ranolazine. Thus, they leave decisions regarding such questions to office-based cardiologists. The majority of interviewed GPs said that they get recommendations and feedback regarding medication from office-based cardiologists.

#### GPs’ perspectives on the impact of NSTEMI, patients’ gender and patients' age on prescribing

None of the GPs reported that they distinguished between NSTEMI and STEMI regarding prescribing:

*‘That’s why in my view there are no big differences in the administration [of drugs], because both involve a corresponding obstruction of vessels and a destruction of tissue [...].’* (GP4, M, B)

Most GPs claimed that neither patients’ gender nor patients’ age matters to them for prescribing after MI:

*‘Primarily it doesn’t matter. So from the approach, [the patient] has a cardiac infarction, gets [medication] now, it remains like that.’* (GP1, F, BB)

The only factor stated which was concerned with patients’ gender was erectile dysfunction as a potential side effect of beta-blockers. A few GPs stated polypharmacy as a reason for prescribing fewer drugs for the elderly after MI. Some GPs argued that older patients might not live long enough to benefit from secondary prevention, while others perceived a higher risk for side effects in older patients:

*‘Very old people, [...] that is the group of those in whom you often have to deal with polypharmacy and prioritisation. [...] Especially in statins the thing is that it is a question of preventive effects, and I personally doubt the preventive effect of a consequent cholesterol reduction in someone who is over 90 [years old].’* (GP14, M, B)

#### Further reasons for not prescribing guideline recommended medication

Initially, half the GPs emphasised that there are no reasons for not prescribing guideline recommended medication after MI. However, all GPs then went on to state at least one reason, most stated several reasons. The most important of these were side effects or intolerances, both of which were named by all GPs:

*‘Well, sometimes under statins one has myopathies, elevations of CK [creatine kinase] or myoglobin or gamma-GT, in case of intolerance, then I discontinue it [...].’* (GP13, F, B)

The majority of the GPs also stated contraindicating comorbidities as reasons for not prescribing, most frequently gastric ulcer contraindicating aspirin. A few GPs claimed guidelines are not adequate to every patient as there are no recommendations for complicating factors such as side effects, comorbidities and polypharmacy.

Further stated reasons for not prescribing the recommended drugs were patients refusing medication, a need for other, more important drugs which can not be given with recommended medication after MI, anticoagulation instead of antiplatelet therapy, palliative situations and patients not tolerating normotonia.

### Non-adherence of patients

#### GPs’ perception of non-adherence

The majority of the GPs reported that only a few of their patients are non-adherent to medication after MI:

*‘Well, I would generally say that the adherence [after MI] is pretty good. Well, here in our area [stated the district] and among our patients.’* (GP6, F, B)

However, some GPs emphasised that non-adherence is the main challenge in the long-term care after MI. Accordingly, some GPs reported finding it laborious to keep trying to convince their patients to take their medication.

#### Assessing medical non-adherence

GPs reported that their methods for assessing medical non-adherence were limited. All GPs check the days covered by the last prescriptions or the intervals between prescriptions:

*‘When I really see that prescriptions really have long intervals, so when there are long intervals, then I ask [the patient] [...].’* (GP14, M, B)

A few GPs explained that they prescribe aspirin, although it is an over-the-counter drug, in order to monitor adherence. Some GPs explained that this checking for prescriptions has its limitations as some patients do not fill their prescriptions or fill them but do not take the medication. Thus, some GPs stated that they ask their patients explicitly in every consultation whether they still take all the drugs in the medication plan. However, this approach only works if patients are honest:

*‘[…] then you ask the patients, “Are you still taking it [the medication] like that?” […]. And I think that then you will figure it out if they are honest.’* (GP12, F, BB)

Some of the interviewed GPs considered unimproved blood pressure or lipid values as indicators of medical non-adherence.

#### Attributed reasons for non-adherence

GPs stated many reasons for non-adherence, which can be sorted into two classes: one concerning patients’ characteristics and the second concerning any other reasons.

One of the most frequently described patient characteristics as a reason for non-adherence was ‘indifference to health’:

*‘Well, you will always have a certain clientele […] who will never follow recommendations, and everyone finds a reason. One [patient] has just watched a telecast, another one says, “I don’t care”.’* (GP3, M, B)

Another GP highlighted indifference to health with ignoring of, carelessness with and disinterest in health:

*‘Well, it is often the case that they [the patients] are incredibly ignoring to their own lives. [...] but, to be honest, it confirms my daily experience that the people are not very careful about their lives. [...] Honestly, I thought that they are so interested in their own health that they come [to me] by themselves. But of course that is not the case.’* (GP2, M, BB)

To highlight indifference to health one GP told of a medical student who did an internship in her surgery:

*‘Well, one [medical student] was somehow really shocked which attitude many people have to their lives. I think that one can’t always imagine this, [...] that there are people who actually have no interest [in their health]. But that is the reality.’* (GP16, F, B)

Additionally, some GPs stated that several patients do not have the capacity to understand why they have to take these drugs:

*‘We have already addressed the topic – let me put it this way now – intellect [of the patient]. That was the only reason [for the patient’s non-adherence]. Indifferent because [he] not at all understood what it is about.’* (GP3, M, B)

Some GPs also stated repression of symptoms and diseases due to fear of further health issues and acute events, and some stated downplaying of diseases:

*‘Well, the discussion about his blood pressure was always like this: [patient:] “I already had a high blood pressure 20 years ago at your father’s [surgery]” – [GP:] “Okay, yes. But that doesn’t mean that it is good that you have a high blood pressure” – “I have always had a high blood pressure”. Like this. It is very difficult to argue with him again and again what the blood pressure does and that it doesn’t matter whether it was already high 20 years ago and blah blah blah. He sees this differently. I would say that he has another world view.’* (GP8, F, B)

A few GPs stated repression of symptoms and diseases and lack of understanding as well as freedom from symptoms also as reasons for patients’ unwillingness to make lifestyle changes including continued smoking. One GP emphasised that non-adherent patients often have had an unhealthy lifestyle, including smoking and alcohol abuse, for their whole lifetime.

Other reasons in the category of patients’ characteristics included aversion to medications, having other priorities, poverty, depression and physical disability.

Almost half of the GPs deemed men less adherent to medication following MI than women and reasoned this with less health consciousness and consequently less attention to and efforts regarding health in men. Accordingly, a few of these GPs reasoned men’s non-adherence with freedom from symptoms and repression of diseases. One GP emphasised that men would deem their MI a weakness. A few GPs also reasoned men’s non-adherence with less diligence and orderliness compared to women.

In addition to patient characteristics, GPs reported further reasons for medical non-adherence. Those reasons are mainly related to side effects and a decreasing subjective need to take the medication over time:

The most important reason is perceived side effects, which lead to autonomous discontinuations by the patients. A few GPs complained that they come to know about these discontinuations some time after they have happened. One GP emphasised that this is often the physicians’ fault:

*‘So the physicians don’t tell the patients, “Well, you get a medication now, it is new to you, it’s called ACE [inhibitor]. Most frequent side effect is dry cough. Don’t discontinue, visit me!”.’* (GP3, M, B)

Some GPs said that adherence following MI depends on the severity of patients’ symptoms and those patients’ acuity as well as the consequently perceived threat to life during MI and on the perception of the acute care, all of which create a frightening situation, which patients do not want to experience again. However, two GPs emphasised that patients’ memories of the MI fade away. One of them said that he reminds his patients of the negative feelings they had experienced during their MI. Another GP emphasised the importance of informing patients about the necessity of the recommended medication right after their MI. However, some GPs claimed that patients get insufficient information about the medication’s effects and necessity in the hospital. Later, due to freedom from symptoms, patients often see no need for continuing with their medication. This reason for non-adherence was stated by half of the GPs:

*‘They would probably say [that] they don’t notice anything [of the medication]. It is not that the [survived] cardiac infarction hurts right now, and when they take the medication they don’t feel better but also not worse.’* (GP5, F, B)

#### Improving adherence

Most GPs regard it as one of their roles to improve the adherence of their patients. GPs use different strategies to improve medical adherence, which can be clustered into four main strategies: preventing side effects, explaining the medication’s necessity, facilitating the intake and involving patients in decision-making.

Almost all GPs stated that they try to prevent side effects before discontinuing the drug causing them, for example by reducing the dose:

*‘Some get problems with their stomach, [...] then you can try reducing to 75 milligrams [of aspirin] for example.’* (GP10, M, BB)

Other strategies include changing substance or drug class, changing distribution of daily doses and prescribing co-medication like proton pump inhibitors.

Another important measure is to explain the medication’s necessity. About half of the GPs do this highlighting the negative consequences of non-adherence, including the chance of dying:

*‘[...] then I say [to the patients], “This is vital for life, aspirin”. […] and then I say, “This is vital because the stent shuts or can shut, and then you are dead, immediately”.’* (GP11, F, BB)

Another GP explained as well that her way of dealing with non-adherence is to threaten with negative consequences:

*‘Then I give him [the patient] a proper talking-to and tell him that he had luck with the cardiac infarction this time, that it was a close shave. It also could have been that he would have got it [the MI] somewhere way out in the sticks. He likes hiking; then he could have got it somewhere on the mountain, there nobody can help him then, and then he bites the dust there. So I rather call a spade a spade [...].’* (GP5, F, B)

The GPs also stated explaining the drug’s effects, drawing analogies, such as calcified pipes, referring to studies, showing risk scores and using visualisation.

The majority of the GPs also stated that they take different strategies to facilitate the intake, such as polypills. However, the majority of GPs was sceptical towards the management of a polypill therapy after MI. A polypill is especially seen as difficult to adhere to since a separation of its components is not possible but sometimes needed due to intolerances or a change of the components’ doses:

*‘Well, that, when side effects occur then, then you can’t discontinue something so easily or reduce or so.’* (GP7, F, BB)

Other stated strategies to facilitate the intake were prescribing nurse-prepared medication, prescribing once-daily doses, discontinuation of less important drugs and recommending pill organisers.

Half of the GPs claimed that they involve patients in decision-making:

*‘So as a GP you have to manage this balancing act [between guidelines and what patients want], that you have to [...], I would say, also make target agreements with the affected person.’* (GP3, M, B)

They highlighted that it is a process of negotiating as the following quote shows:

*‘He [the patient] noted sometime that these panic attacks and this discomfort [...], that this doesn’t appear that often when he omits the beta-blocker. He discontinued it, and then I said, “Well, but take a half dose at least, see how it goes then”. And we sort of negotiated about it.’* (GP9, M, BB)

The majority of the interviewed GPs emphasised that ultimately, the patients have to make decisions and are responsible for their health. Accordingly, the majority explained that they accept medical non-adherence after they have tried to convince their patients:

*‘Then I often say, “However, to my father I would administer it now, would insist that he takes it”. And if they [the patients] don’t take it then, it will be their decision.’* (GP12, F, BB)

## Discussion

In this article, we examined the GPs’ perspective on long-term care after MI regarding prescribing and medical non-adherence following MI. Almost all GPs reported that they follow guidelines independently of patients’ type of MI and gender and that they continue to prescribe the medication that was initiated in the hospital. Some GPs reported uncertainties regarding DAPT and triple therapy and that office-based cardiologists support them in such cases. Main reasons for not prescribing guideline-recommended medication were side effects and intolerances and comorbidities. A few GPs claimed lacking recommendations for these factors and for polypharmacy. Some GPs question the effects of secondary prevention for the oldest of patients.

The interviewed GPs perceived good adherence to secondary preventive medication among their patients who had had an MI while they regarded their methods for assessing medical non-adherence as limited.

The GPs perceived diverse reasons for non-adherence, particularly side effects, patients’ freedom from symptoms and indifference to health. Another important reason was patients’ lack of knowledge and understanding. Some GPs claimed that patients get insufficient information about the medication in the hospital.

The GPs’ strategies for improving adherence include preventing side effects, explaining the necessity of the medication, facilitating intake and involving patients in decision-making. About half of them reported threatening their patients with negative consequences of non-adherence.

### Comparison with existing literature

Our findings relating to GPs’ perspective on reasons for not prescribing recommended medication following MI are in accordance with quantitative findings after MI [20–22] and qualitative findings in statin therapy [23–25]. The fact that guidelines rarely address complicating factors such as comorbidities [32] was claimed by GPs in our study too. However, contrary to a few GPs’ opinion, recent guidelines for STEMI and for NSTEMI by the European Society of Cardiology [7, 9] and by the American Heart Association and the American College of Cardiology [8, 10] do address side effects or intolerances and contraindicating comorbidities. However, they do not address multimorbidity and consequential polypharmacy [7–10].

In contrast to studies showing lower prescribing after NSTEMI [19, 26] and in women [15, 27], GPs negated prescribing differently in these sub-groups. It is already known that discharge medication prescribed by the hospital after an MI is insufficient [19, 42]; however, interviewed GPs stated that they usually adhere to these recommendations. A possible reason for this non-guideline recommendation in discharge letters might be the older age of NSTEMI patients [19, 42] and female MI patients [12, 43].

Our findings regarding GPs’ perspective on reasons for not prescribing recommended medication in older patients correspond with qualitative findings for statins [23, 36] and post-MI prescribing in the elderly [31]. However, in contrast to a focus group study from the Netherlands, our study has not revealed barriers relating to organisation, such as the finding that many patients had fallen into the gap between secondary and primary care and were no longer visiting their specialist [31].

GPs perceived good adherence to secondary preventive medication following MI. This is in contrast to a study which has shown that adherence after MI is insufficient [33] and underlines that their adherence assessment is limited. That physicians assess non-adherence by checking prescription refill records [36], by asking the patients [44, 45] and by monitoring lipid values [36] has also been reported in previous studies. Furthermore, GPs’ view that adherence depends on patients’ experiences during MI is in accordance with findings from interviews with patients after MI [46].

GPs perceived side effects and patients’ freedom from symptoms as reasons for non-adherence. These perceptions have also been reported by GPs in studies which have addressed statin therapy [23, 25]. Some GPs also perceived indifference to health as a reason for non-adherence, and they mainly described it as a character trait. In line with this, a previous study found that patients with greater concern about heart health were more open to statins’ benefits [25]. However, to our knowledge, indifference to health as a reason for non-adherence has not been reported before in the literature, although most of the reasons for non-adherence perceived by the GPs in this study are in accordance with those reported by patients in previous studies [25, 34, 37, 47–49]. This implies that indifference to health might be only the GPs’ perception. What GPs perceive as indifference to health might in fact be a functional behaviour of the patient to try to reduce ambivalence and fear, such as coping-behaviour. Another explanation might be that some patients have other values and priorities than the GPs, which might be perceived as indifference to health. Further explanations might be an external health locus of control [50], low self-efficacy [51] and patients’ freedom from symptoms.

The reported characteristics of non-adherent patients, such as patients’ lack of knowledge and understanding, were mainly negative and promote the laboriousness of convincing these patients. This indicates a helplessness of the GPs in the management of these patients.

One potential method for exploring underlying motives and motivations is motivational interviewing [52]. Motivational interviewing can improve adherence, but studies demonstrating this in the context of medical adherence in cardiovascular diseases are scarce [52, 53]. Accordingly, none of the GPs mentioned the use of motivational interviewing, although we explicitly asked them about their strategies for improving adherence. This is in accordance with the finding that GPs who were working in Berlin, Germany, showed a low to moderate use of motivational interviewing techniques during individual risk counselling with overweight or obese patients during which any medical problem requiring a decision occurred [54]. The interviewed GPs might use techniques of motivational interviewing without naming it. However, they reported to confront their patients with the danger of death and expected ratio-based interventions such as education to be successful, which contradicts the approach of motivational interviewing.

Physicians’ reported strategies for improving adherence through preventing side effects [25, 36], education [24, 25, 36, 45, 55], reduced frequency of administration per day [45] and involving patients in decision-making [36, 56] are reflected in previous studies into statin therapy [24, 25, 36], CHD prevention [55], diabetes mellitus [45] and hypertension [45, 56]. German GPs and internists had positive attitudes towards combination pills for hypertension [57] and type 2 diabetes [58]; however, our interviewed GPs criticised the inflexible management of polypills after MI.

### Strengths and limitations

To our knowledge, this is the first comprehensive qualitative study which examined the prescribing of and non-adherence to secondary preventive medication following MI from the perspective of GPs.

Recruitment strategies might have selected GPs who are better informed and more knowledgeable about this area than German GPs on average, and participating GPs might have been more interested in the study’s topics and consequently more knowledgeable about them than non-participating GPs.

Our results are not statistically representative due to the qualitative approach and limited number of interviewees. However, saturation regarding the topics and themes has been reached. In addition, the sample has a wide variety regarding the GPs’ age and the number of GPs in each surgery. The distribution of genders is nearly equal and the surgeries were located in both urban and rural regions. Furthermore, Germany had been divided into two states until 1990, and the surgeries of our study were located in both former East Germany, including former East Berlin, and former West Berlin, which had been aligned to former West Germany. Moreover, the German healthcare system is mainly based on federal law. Thus, we assume that the results are mainly generalisable to GP care in Germany.

The high number of GPs reporting that they adhere to guidelines points to social desirability effects, which are balanced by the prompted case narratives.

## Conclusions

Our results highlight that guidelines should explicitly address multimorbidity and consequential polypharmacy. They should provide decision aids which take account of patients’ sex, age, risk factors and comorbidities so that GPs and patients can make a better-informed prioritisation of diseases to treat and drugs to use. Decision-aid software could facilitate these individual prioritisations and could visualise risks and risk reductions for patients’ understanding [25].

GPs seem to often rely on hospitals’ discharge medication. Because of the already known insufficient discharge medication after MI this can lead to insufficient medical secondary prevention. Thus, the discharge recommendations should be checked for accordance with the guidelines in the hospital, for example automatically by software, and GPs should be aware of this problem and should also perform such checks.

GPs partly question the effects of secondary prevention for the oldest of patients. Future research should address which of the oldest of patients benefit from medical secondary prevention after MI and which do not. Results of such studies could help GPs and patients to assess the benefit from medical secondary prevention and enable a better-informed decision-making.

In daily practice, GPs should talk with patients who had an MI about adherence and long-term treatment goals including their perspective on health. GPs should assess whether patients are indifferent to their health or whether there are underlying reasons for non-adherence which only mimic indifference to health. A trustful partnership may be an important resource, and it may be helpful to assess patients’ motivations through motivational interviewing to understand this distinction. Further studies should address indifference to health explicitly and its possible association with repression of health issues, health literacy, health locus of control and self-efficacy. Improving physicians’ communication skills and informing and motivating patients in an adequate manner, for example in simple language, should be an important goal and should already start in the hospital. To improve communication skills, training in motivational interviewing during studies, training and continuous education might be a possible measure.

## Supplementary information

**Additional file 1.** Interview guide. Final version of the interview guide, translated from German into English.

**Additional file 2.** Major topics, subthemes and subcodes. List showing the two major topics with their subthemes and the subthemes’ subcodes.

## Data Availability

The datasets generated and analysed during the current study are not publicly available due to participant and patient confidentiality.

## Abbreviations

ACEI: Angiotensin converting enzyme inhibitors
B: Berlin
BB: Brandenburg
CHD: Coronary heart disease
DAPT: Dual antiplatelet therapy
F: Female
GP: General practitioner
M: Male
MI: Myocardial infarction
NSTEMI: Non-ST-elevation myocardial infarction
STEMI: ST-elevation myocardial infarction
