# Supplementary material for: Prescribing and medical non-adherence after myocardial infarction: qualitative interviews with general practitioners in Germany
Source: BMC Fam Pract. 2020 May 8;21:81. doi: 10.1186/s12875-020-01145-6 (PMC7210678; doi:10.1186/s12875-020-01145-6)
Supplement: Supplementary file 1 — Additional file 1. Interview guide. Final version of the interview guide, translated from German into English. [file 12875_2020_1145_MOESM1_ESM.pdf]

Prescribing and medical non-adherence after myocardial infarction: qualitative interviews with general practitioners in Germany

Christian Freier, Christoph Heintze and Wolfram J. Herrmann

BMC Family Practice (2020)

Additional file 1: Interview guide

Final version, translated from German into English

Questions which were added to the guide after the transcription of an interview are written in italics and commented with the number of the interview to which they were applied for the first time.

Introduction

- We are particularly interested in your experiences from your daily practice and also in your subjective perceptions. Thus, it is definitely desired that you tell about experienced situations and particularise them.
- When I say ‘cardiac infarction’, I mean both ST-elevation myocardial infarction (STEMI) and non-ST-elevation myocardial infarction (NSTEMI). When the focus is only on one of these forms I will say that explicitly.

Warm-up

- Please tell me about one of your last consultations with a patient who had a cardiac infarction some time ago.
- Can you tell me about another case, which had a different course?

Role of the general practitioners

- In which role do you see yourself as a general practitioner in the long-term

care after cardiac infarction?

  - What are your tasks?

- Which challenges are you facing as a general practitioner in the long-term care after cardiac infarction and in what way?
- What could be improved in the long-term care after cardiac infarction and how?

Drug treatment after cardiac infarction (deficits, adherence, differences due to gender and age)

- Which drugs are you prescribing to the initially mentioned patient because of the cardiac infarction?
  - Which other drugs are you prescribing to patients after cardiac infarction and why?
  - Which drugs do you prefer and why?
  - Which drugs do you rather dislike and why?
- For each recommended drug class after cardiac infarction (angiotensin converting enzyme inhibitors / angiotensin receptor blockers, beta-blockers, aspirin, $P2Y_{12}$ inhibitors, statins) the percentage of women and the percentage of men who had a prescription of the respective drug group filled in the fourth quarter after their cardiac infarction was under 65 % [1]. This can be due to physicians' non-prescribing or patients' non-adherence.

  Please tell me about one of your last patients who has been consulting you and had had a cardiac infarction some time ago and to whom you did not prescribe one or more of these drugs.
  - What were the reasons?
  - Are there further reasons that you would not prescribe these drugs after cardiac infarction? Which reasons?
  - Are you prescribing these drugs differently depending on gender and age or independently of these factors after cardiac infarction?

        - If prescribing differently: In what way and why?
        - And how is it with statins after cardiac infarction?
    - Are you prescribing differently after an NSTEMI compared to a STEMI or are you prescribing in the same way? In what way and why?

- Did it seem to you that the mentioned patient did not take his/her drugs as prescribed? (Or: Please tell me about one of your last patients who has been consulting you and had had a cardiac infarction some time ago and who seemed to you as he/she did not take the drugs as prescribed.)
    - What do you think were his/her reasons?
    - What do you think are further reasons that patients do not take the drugs as prescribed after cardiac infarction?
    - How big is the extent of medical non-adherence in your patients after cardiac infarction?
    - How are you dealing with medical non-adherence after cardiac infarction?
    - From your daily experience are there drugs to which the adherence is particularly low compared to other drugs after cardiac infarction?
        - What do you think are the reasons?
    - From your daily experience in what way are there differences in the adherence after STEMI and NSTEMI or are there no differences?
        - If there are differences: What do you think are the reasons?
    - What do you think how the medical adherence after cardiac infarction could be improved?
        - *What do you think about the polypill, which contains several agents in one pill? [for the first time applied to the sixth interview]*
            - *What do you think are the advantages and disadvantages? [for the first time applied to the sixth interview]*

        - *Which role does the disease management programme for patients with coronary heart disease play for you in the cardiac infarction aftercare? [for the first time applied to the tenth interview]*
            - *Which patients are you including in this disease management programme after cardiac infarction? [for the first time applied to the tenth interview]*
            - *What do you think about this disease management programme? [for the first time applied to the tenth interview]*
- How did you notice that this patient does not take the drugs as prescribed?
    - Are you asking your patients explicitly about their medical adherence?
        - If yes: In which cases are you doing this?
        - If yes: How are the patients reacting then?
        - If not: Why not?
- The percentage of older women after cardiac infarction who had prescriptions of statins filled is lower than the corresponding percentage of older men [1]. What are your perceptions of the prescribing of statins and of the adherence to statins in older women after cardiac infarction?
    - How are you dealing with this issue I just described?
    - Are you focusing particular attention on the prescribing of statins and on the adherence to statins in older women after cardiac infarction?
- The percentage of younger women after cardiac infarction who had prescriptions of aspirin filled is lower than the corresponding percentage of older men [1]. What do you think are the reasons? (regarding both prescribing and adherence)
    - What are your perceptions of the prescribing of aspirin and of the adherence to aspirin in younger women after cardiac infarction?

  - How are you dealing with this issue I just described?
  - Are you focusing particular attention on the prescribing of aspirin and on the adherence to aspirin in younger women after cardiac infarction?

- *Can you please tell me about a patient after cardiac infarction who takes two antiplatelet drugs and in addition has an indication for oral anticoagulation? [for the first time applied to the seventh interview]*
  - *What are your experiences with such situations? [for the first time applied to the seventh interview]*
  - *How are you dealing with them? [for the first time applied to the seventh interview]*

<u>Coronary heart disease in the context of multimorbidity</u>

- In most cases a cardiac infarction is caused by coronary heart disease. Patients with coronary heart disease are often at an age in which they also have other chronic diseases, aside from coronary heart disease risk factors such as diabetes or hypertension. For you as a general practitioner is the coronary heart disease particularly important compared to other chronic diseases, or is it just one of many diseases or are there more important diseases? Why?
- Please tell me about one of your last patients who has been consulting you and has a coronary heart disease and another disease which influences or has influenced the treatment of the coronary heart disease.
  - Which further diseases influence the treatment of the coronary heart disease and in what way?
- Please tell me about one of your last patients who has been consulting you and had had a cardiac infarction some time ago whose combination of diseases is particularly difficult for you as a general practitioner.
  - In what way was this combination of diseases difficult for you?
  - Which other combinations of diseases in patients after cardiac

infarction are particularly difficult for you as a general practitioner and in what way?

  - What are your strategies for dealing with these combinations?

- I was surprised that mental issues and illnesses are not uncommon after cardiac infarction (e.g. depression, anxiety disorders, posttraumatic stress disorder). What are your perceptions of this issue in your surgery?
  - How are you dealing with this? (screening, diagnosis, therapy)

<u>Collaboration with cardiologists and other healthcare services</u>

- Could you please tell me about one of your last referrals of a patient who had had a cardiac infarction some time ago to a cardiologist?
  - In which further situations do you refer patients who had had a cardiac infarction some time ago to a cardiologist and why?
  - Could you please tell me about one of your last of such referrals, which you were asked for by the patient?
    - What was his/her reason? What are further reasons?
    - What do you think and how do you feel when a patient asks you for such a referral?
    - On what does it depend whether you refer in such situations or not?
    - *Are there patients who do not want to go to the cardiologist after cardiac infarction? [for the first time applied to the seventh interview]*
      - *What are their reasons? [for the first time applied to the seventh interview]*
- Could you please describe the process of collaboration and communication with the cardiologist in the context of the mentioned referral?

  - How would you rate the collaboration and communication and why?
  - *Are you getting medical reports from the cardiologists? [for the first time applied to the seventh interview]*
  - What do you think are the difficulties in the collaboration and communication, and what are your strategies for dealing with them?
  - What do you think how the collaboration and communication could be improved?
- Let's say that patients after cardiac infarction could consult office-based cardiologists only with a referral by their general practitioner. What would you think about such a regulation and why?
  - What do you think would be the advantages and disadvantages of such a regulation?
- Let me show you cards with different healthcare services (cardiac rehabilitation exercise classes, nutrition counselling, support groups, psychosocial care, nursing). Which of these have you already recommended for patients who had had a cardiac infarction some time ago?
  - Are there any other healthcare services you already have recommended for patients who had had a cardiac infarction?
  - In which cases are you recommending [healthcare services mentioned by interviewee] after cardiac infarction and why in these cases?
  - Why are you not recommending [healthcare services not mentioned by interviewee]?
  - What are the difficulties with [healthcare services mentioned by interviewee], and what are your strategies for dealing with these difficulties?
    - Where do you see potential for improvements?
  - *What do you think which further healthcare services would be necessary in addition? [for the first time applied to the eighth interview]*
- *What do you recommend your patients for smoking cessation? [for the first*

time applied to the eighth interview]

  - *What are the difficulties? [for the first time applied to the eighth interview]*

- *What do you think about rehabilitation programmes after cardiac infarction? [for the first time applied to the sixth interview]*
  - *To which patients are you recommending such programmes? [for the first time applied to the sixth interview]*
  - *Is there any room for improvements? [for the first time applied to the sixth interview]*
  - *Did any of your patients deny rehabilitation programmes after cardiac infarction? [for the first time applied to the seventh interview]*
    - *What were their reasons? [for the first time applied to the seventh interview]*
  - *Do you think rehabilitation programmes after cardiac infarction should be performed in stationary or ambulatory conditions and why? [for the first time applied to the twelfth interview]*

Conclusion

Which important topics of long-term care after cardiac infarction have been missing in the interview?

Reference

1. Ulrich R, Pischon T, Robra B, Freier C, Heintze C, Herrmann W. Health care utilisation and medication one year after myocardial infarction in Germany – a claims data analysis. Int J Cardiol. 2020;300:20-26
