# Supplementary material for: Prescribing and medical non-adherence after myocardial infarction: qualitative interviews with general practitioners in Germany
Source: BMC Fam Pract. 2020 May 8;21:81. doi: 10.1186/s12875-020-01145-6 (PMC7210678; doi:10.1186/s12875-020-01145-6)
Supplement: Supplementary file 2 — Additional file 2. Major topics, subthemes and subcodes. List showing the two major topics with their subthemes and the subthemes’ subcodes. [file 12875_2020_1145_MOESM2_ESM.pdf]

Prescribing and medical non-adherence after myocardial infarction: qualitative interviews with general practitioners in Germany

Christian Freier, Christoph Heintze and Wolfram J. Herrmann

BMC Family Practice (2020)

Additional file 2: Major topics, subthemes and subcodes

Major topic 1: Prescribing of medication after myocardial infarction

- Prescribed medications
    - Angiotensin converting enzyme inhibitors, angiotensin receptor blockers
    - Aspirin
    - Beta-blockers
    - Statins
- Hospital recommendations
    - Calling the hospital in case of inconsistencies in the discharge letter
    - Continuing the medication initiated in the hospital
    - General practitioner (GP) would not adopt hospitals' recommendations if they were nonsense
    - Including information about every patient's prior medication on the admission form
    - Patients discharged on medication which has already been proven to be inappropriate for them
    - Trust in inpatient cardiologists
- Cardiologists' role in GPs' prescribing

- Cardiologists decide about dual antiplatelet therapy, triple therapy, sacubitril/valsartan combination or ranolazine
- Cardiologists give recommendations and feedback regarding medication
- For GP hard to keep track of the recommendations for dual antiplatelet therapy
- For GP hard to keep track of the recommendations for triple therapy
- GP prescribes medication him-/herself
- GP's inexperience in prescribing sacubitril/valsartan combination or ranolazine

- GPs' perspectives on the impact of non-ST-elevation myocardial infarction, patients' gender and patients' age on prescribing
  - Not distinguishing between non-ST-elevation myocardial infarction and ST-elevation myocardial infarction
  - Role of patients' gender
    - Erectile dysfunction as a potential side effect of beta-blockers
    - Patients' gender does not matter for prescribing after myocardial infarction
  - Role of patients' age
    - Patients' age does not matter for prescribing after myocardial infarction
    - Reasons for not prescribing recommended drugs after myocardial infarction in older patients
      - Higher risk for side effects in older patients
      - Older patients might not live long enough to benefit from secondary prevention
      - Polypharmacy
- Further reasons for not prescribing guideline recommended medication

  - Anticoagulation instead of antiplatelet therapy
  - Contraindicating comorbidities
  - Need for other, more important drugs which can not be given with recommended medication after myocardial infarction
  - No reasons
  - Normal lipid values (regarding statins)
  - Palliative situation
  - Patient does not tolerate normotonia
  - Patient refuses medication
  - Side effects or intolerances

Major topic 2: Non-adherence of patients

- GPs' perception of non-adherence
  - Laboriousness of trying to convince patients
  - Non-adherence as main challenge in long-term care after myocardial infarction
  - Only few patients non-adherent to medication after myocardial infarction
- Assessing medical non-adherence
  - Asking patients explicitly whether they still take all the drugs in the medication plan
  - Checking days covered by the last prescriptions or the intervals between prescriptions
  - Methods are limited
    - Asking explicitly will only work if patients are honest
    - Some patients do not fill prescriptions

        - Some patients do not take the medication
    - Prescribing aspirin, although it is available without a prescription, in order to check the days covered or intervals
    - Unimproved blood pressure or lipid values as indicators
- Attributed reasons for non-adherence
    - Characteristics of non-adherent patients after myocardial infarction
        - Alcohol abuse
        - Always busy with the job
        - Aversion to medication
            - Aversion to sudden increase in number of medications
            - Aversion to long-term medication
            - Aversion to taking too many medications
            - General aversion to any medication
        - Depression
        - Downplaying of diseases
        - Expecting soon death because of high age
        - Fear of side effects
        - Indifference to health
        - Lack of intellectual capabilities
        - Low socioeconomic status
        - Male gender
            - Deeming myocardial infarction a weakness
            - Freedom from symptoms
            - Less attention to health than women
            - Less diligence than women

      - Less efforts regarding health than women
      - Less health consciousness than women
      - Less orderliness than women
      - Repression of disease
    - Patient did not perceive his/her myocardial infarction life-threatening
    - Patient has only prescriptions of familiar drugs filled
    - Patient perceives other problems as more important
    - Physical disability
    - Poverty
    - Repression of symptoms and diseases due to fear of further health issues and acute events
    - Smoking
    - Unwillingness to make lifestyle changes
  - Further reasons for medical non-adherence following myocardial infarction
    - Adherence decreases with increasing number of medications
    - Belief that medications on over-the-counter prescriptions are herbal, since over-the-counter prescriptions are green*
    - Discontinuing antihypertensives when blood pressure target is reached
    - Forgetting the intake
    - Freedom from symptoms
    - Medications' low prices cause perception of low necessity and efficacy
    - Memory of myocardial infarction fades away

        - Patient forgets to buy aspirin because the corresponding over-the-counter prescription does not have to be given to the pharmacist*
        - Patient not involved in decision-making
        - Pharmacy dispensed medication of different brand and look
        - Poor to no explanation of newly prescribed medications' effects and necessity in the hospital
        - Side effects
        - Unawareness of the medication's limitless duration
- Improving adherence
    - Explaining the necessity of the medication
        - Drawing analogies such as calcified pipes
        - Explaining the consequences of non-adherence
        - Explaining the drug's effects
        - Referring to studies
        - Showing risk scores
        - Using visualisation
    - Facilitating the intake
        - Prescribing nurse-prepared medication
        - Prescribing once-daily doses
        - Recommending pill organisers
        - Reducing the number of pills through polypills or discontinuation of less important drugs
    - Involving patients in decision-making
    - Preventing side effects before discontinuing the causing drug
        - Changing distribution of daily doses

- Changing to other substances or other drug classes
- Prescribing co-medication (e.g. proton pump inhibitors)
- Reducing the dose

* In Germany physicians can write over-the-counter prescriptions for non-prescription drugs, which also include most herbal drugs. Over-the-counter prescriptions are green and serve as reminders for the patients and for the pharmacists' information, if necessary. In contrast to prescriptions for prescription drugs, over-the-counter prescriptions do not have to be given to the pharmacists in order to buy non-prescription drugs.
